# Encoding gene RAB3B exists in linear chromosomal and circular extrachromosomal DNA and contributes to cisplatin resistance of hypopharyngeal squamous cell carcinoma via inducing autophagy

**DOI:** 10.1038/s41419-022-04627-w

**Published:** 2022-02-22

**Authors:** Changwei Lin, Yifei Chen, Fan Zhang, Baiying Liu, Canbin Xie, Yexun Song

**Affiliations:** 1 Department of Gastrointestinal Surgery, The Third Xiangya Hospital of Central South University, Changsha, 410013 Hunan Province China; 2 School of Life Sciences, Central South University, Changsha, 410078 Hunan Province China; 3 Department of Otolaryngology-Head Neck Surgery, The Fourth Hospital of Changsha (The Changsha Affiliated Hospital of Hunan Normal University), Hunan Normal University, Changsha, 410006 Hunan Province China; 4 Department of Otolaryngology-Head Neck Surgery, The Third Xiangya Hospital of Central South University, Changsha, 410013 Hunan Province China; 5 Department of Otolaryngology-Head Neck Surgery, The Xiangya Hospital of Central South University, Changsha, 410008 Hunan Province China

**Keywords:** Head and neck cancer, Cancer screening, DNA recombination

## Abstract

Cisplatin (DDP) resistance is an important factor that decreases the effect of chemotherapy, thus leading to local recurrence and lymph node metastasis of hypopharyngeal squamous cell carcinoma (HSCC). We aimed to explore the role and mechanism of extrachromosomal circular DNA (eccDNA) in the DDP resistance of HSCC. In our research, the HSCC cell line FaDu and the DDP-resistant cell line FaDu/DDP were used as subjects. eccDNA sequencing and whole transcriptome sequencing were conducted, followed by a combined analysis of the two sequencing profiles. Outward PCR, inward PCR and Sanger sequencing were used to verify sequences of the eccDNAs. Bioinformatics analysis based on TCGA/GEO was performed in addition to plasmid transfection, RNA interference, qRT-PCR and Western blot experiments to verify the expression level of RAB3B amplified from eccDNA. mRFP-GFP-LC3 adenoviral particle transfection and transmission electron microscopy were used to detect autophagic flux. Finally, we evaluated the role of RAB3B in FaDu/DDP cells and patient-derived organoids. Our results showed that we purified and sequenced more than 10 thousand eccDNAs from the two cell lines, and the size of the eccDNAs was distributed from 0.01 kb to 1000 kb. The combined analysis between eccDNA and transcript sequencing indicated that there were some highly expressed genes that were completely or partially transcribed from related sequences of eccDNAs and not from genome linear DNA. We further screened and verified the encoding gene RAB3B using full-length sequences that might be amplified from eccDNA [*chr1*^*circle 46219-52682 kb*^]. Finally, we confirmed that RAB3B could promote DDP resistance in HSCC by inducing autophagy. The eccDNA might play significant roles in DDP resistance in HSCC by amplifying related functional genes. Further study is needed to explore the novel mechanisms of eccDNA in the drug resistance of HSCC.

## Introduction

Extrachromosomal circles of DNA (eccDNA) are various sizes of circular DNA ranging from hundreds to thousands of base pairs (bp) that are found within a preparation of mammalian DNA [1] and have been proven to participate in physiological [2] or pathological [3] processes in a special way. Considering that oncogene amplification is one of the common types of cancer mutations, an increasing number of studies have focused on eccDNAs and found that they [4] play a similar role in oncogene amplification as chromosomes [5]. For example, Wu S et al. proved that oncogenes encoded on eccDNAs are among the most highly expressed genes in the transcriptome of tumors, thus linking increased copy numbers with high transcription levels [6]. Zhu Y et al. found that there are a number of eccDNAs in prostate cancer that can function as mobile transcriptional enhancers to promote tumor progression [7]. Furthermore, eccDNAs have also been frequently found in 29 cancers, including head and neck squamous cell carcinoma (HNSCC) [8].

Hypopharyngeal squamous cell carcinoma (HSCC) has the worst prognosis among HNSCCs and is rapidly rising in incidence [9]. Approximately 70–85% of HSCCs are diagnosed at stage III or IV, and the 5-year overall survival rate is less than 45% [10]. Cisplatin (DDP)-based chemotherapy is an important treatment to improve the prognosis of HNSCC [11]. However, the high incidence of drug resistance has become an important factor affecting the effect of chemotherapy, thus leading to local recurrence and consequent lymph node metastasis [12]. Therefore, identifying the cellular and molecular mechanisms of DDP resistance is helpful for developing effective therapies. Mounting evidence shows that eccDNAs also play an important role in drug resistance. For example, D. H. Koo found that eccDNA-based amplification and transmission are key to obtaining herbicide resistance in *Amaranthus palmeri* [13]. In addition, eccDNA encompasses the pteridine reductase-1 gene (PTR1) responsible for MTX resistance [14]. These results suggest that oncogene amplification of eccDNAs is an important method by which tumor cells develop chemoresistance. However, the expression profiles of eccDNA in HSCC are still unclear, and whether there is a specific eccDNA devoted to DDP resistance in HSCC has not been well studied.

Here, we first investigated the different expression profiles of eccDNAs between FaDu/DDP cells and FaDu cells. Then, we confirmed that eccDNAs are derived from every human chromosome with sequences from all known types of genomic structures, including genes and intergenic and repetitive regions, thus revealing that eccDNAs are common mutational elements in HSCC. Finally, we identified that eccDNA could promote FaDu/DDP cell resistance to DDP by amplifying the RAB3B gene, which can enhance the autophagy of FaDu/DDP cells. Our discovery suggests that producing more eccDNAs is an important method used by FaDu cells to induce and promote DDP resistance. Understanding the tolerance mechanisms may provide new strategies for HSCC therapy.

## Methods and materials

### eccDNA enrichment for circle-Seq

eccDNA enrichment, purification and sequencing were performed as described in a previous study [2]. Briefly, cells were suspended in L1 solution (Plasmid Mini AX; A&A Biotechnology) and supplemented with Proteinase K (Thermo Fisher) before incubation overnight at 50 °C with agitation. After lysis, the samples were treated with an alkaline solution, followed by the precipitation of proteins and separation of chromosomal DNA from circular DNA through an ion exchange membrane column (Plasmid Mini AX; A&A Biotechnology). Column-purified DNA was treated with FastDigest MssI (Thermo Scientific) to remove mitochondrial circular DNA and incubated at 37 °C for 16 h. The remaining linear DNA was removed by exonuclease (Plasmid-Safe ATP-dependent DNase, Epicentre) at 37 °C in a heating block, and the enzyme reaction was carried out continuously for 1 week, with additional ATP and DNase added every 24 h (30 units per day) according to the manufacturer’s protocol (Plasmid-Safe ATP-dependent DNase, Epicentre). eccDNA-enriched samples were used as templates for phi29 polymerase amplification reactions (REPLI-g Midi Kit) that amplified eccDNA at 30 °C for 2 days (46–48 h). Phi29-amplified DNA was sheared by sonication (Bioruptor), and the fragmented DNA was subjected to library preparation with the NEBNext^®^ Ultra II DNA Library Prep Kit for Illumina (New England Biolabs). Sequencing was carried out on an Illumina NovaSeq 6000 with 150 bp paired-end mode according to the manufacturer’s instructions. The Circle-Seq eccDNA sequencing service was provided by CloudSeq Biotech Inc. (Shanghai, China).

### Data analysis

Paired-end reads were harvested from an Illumina NovaSeq 6000 sequencer and quality controlled by Q30. After 3’ adaptor trimming and low-quality read removal by cutadapt software (v1.9.1), the high-quality clean reads were aligned to the reference genome (UCSC hg19) with bwa software v (v0.7.12). Then, circle-map software (v1.1.4) was used to detect eccDNA within all samples, and SAMtools (v0.2) software was used to obtain raw soft-clipped read counts of the break point. Then, edgeR (v0.6.9) software was used to perform normalization and differentially expressed eccDNA filtering based on the p-value and fold change. The software bedtools (v2.27.1) was used to annotate the eccDNAs. GO and pathway enrichment analyses were performed based on the differentially expressed eccDNA-associated genes. IGV (v2.4.10) software was used for eccDNA visualization.

### Validation of eccDNA recordings

Outward directing PCR oligos were designed in Primer3web (v4.0) and devised to yield products across junctions of 4 detected circular DNA structures (Supplementary Table 11). Each 10 µl PCR typically included 120 ng phi29-amplified template (2 µL), 10 µM primer, and 2 × Master Mix (5 µL), and the PCR assay was performed for 40 cycles in a PCR cycler under standard PCR conditions. All reactions were performed with controls for human genomic DNA. Inward-designed oligos (Supplementary Table 11) represented positive controls for PCRs with circular and linear DNA templates. Size separation of PCR products based on agarose (0.6–1.5%) gel electrophoresis and Sanger sequencing of PCR products confirmed the circular structure of selected eccDNAs.

### Statistical analysis

All values are expressed as the mean ± standard deviation (SD). The significance of differences was determined by one-way ANOVA or Student’s *t* test, and a value of *p* < 0.05 was considered significant. Statistical analyses were performed using SPSS (v20.0).

## Results

### Genome-wide detection of eccDNAs in FaDu/DDP and FaDu cells

**Fig. 1:**
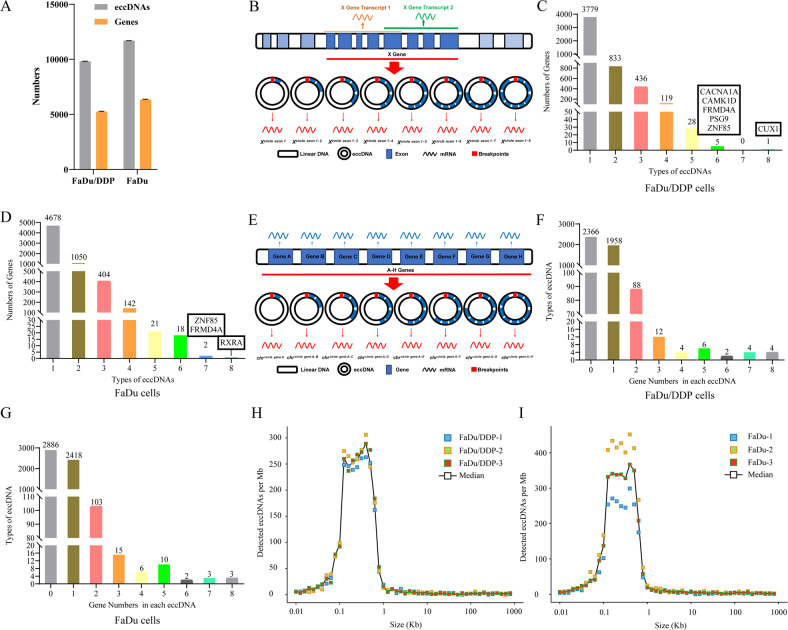
Genome-wide detection of eccDNAs in FaDu/DDP and FaDu cells. **A** Number of eccDNA types and involved genes from FaDu/DDP and FaDu cells are depicted according to the cell lines. **B** Schematic representation of the mechanism of different types of eccDNA amplifying the same gene. **C** Number of genes derived from 1, 2, 3, 4, 5, 6, 7 or 8 different types of eccDNAs in FaDu/DDP cells. **D** Number of genes derived from 1, 2, 3, 4, 5, 6, 7 or 8 different types of eccDNAs in FaDu cells. **E** Schematic representation of the mechanism by which different types of eccDNAs amplify more than one gene. **F** Number of eccDNA types amplifying 1, 2, 3, 4, 5, 6, 7 or 8 different genes in FaDu/DDP cells. **G** Number of eccDNA types amplifying 1, 2, 3, 4, 5, 6, 7 or 8 different genes in FaDu cells. **H** Size of eccDNA ranged from 0.01 kb to 1000 kb in FaDu/DDP cells. **I** Size of eccDNA ranged from 0.01 kb to 1000 kb in FaDu cells.

To obtain knowledge about eccDNAs in FaDu/DDP and FaDu cells, we adapted Circle-Seq to detect eccDNAs on a genomic scale. As the results showed, we identified 9773 eccDNAs and 5201 eccDNA-amplified genes in FaDu/DDP cell samples (Fig. 1A, Supplementary Table 1) and identified 11688 eccDNAs and 6316 eccDNA-amplified genes in FaDu cell samples (Fig. 1A, Supplementary Table 2).

According to the generation mechanism, the eccDNAs could map to some or all the exons of certain protein-coding genes (Fig. 1B). Among the FaDu/DDP cell lines, we found that there were 3779 genes derived from only one type of eccDNA, 833 genes derived from 2 types of eccDNAs, 436 genes derived from 3 types of eccDNAs, 119 genes derived from 4 types of eccDNAs, 28 genes derived from 5 types of eccDNAs, 5 genes derived from 6 types of eccDNAs and only one gene (CUX1) derived from 8 types of eccDNAs (Fig. 1C). Among the FaDu cell lines, we found that there were 4678 genes derived from only one type of eccDNA, 1050 genes derived from 2 types of eccDNAs, 404 genes derived from 3 types of eccDNAs, 142 genes derived from 4 types of eccDNA, 21 genes derived from 5 types of eccDNAs, 18 genes derived from 6 types of eccDNAs, 2 genes derived from 7 types of eccDNAs, and only one gene (RXRA) derived from 8 types of eccDNAs (Fig. 1D).

In addition, eccDNAs amplified different types of encoding genes (Fig. 1E). During the FaDu/DDP cell, we found that there were 2366 types of eccDNAs did not amplify any genes, 1958 types of eccDNAs amplified 1 gene, 88 types of eccDNAs amplified 2 genes, 12 types of eccDNAs amplified 3 genes, 4 types of eccDNAs amplified 4 genes, 6 types of eccDNAs amplified 5 genes, 2 types of eccDNAs amplified 6 genes, 4 types of eccDNAs amplified 7 genes, and 4 types of eccDNAs amplified 8 genes (Fig. 1F). Among the FaDu cell lines, we found that 2886 types of eccDNAs did not amplify any genes, 2418 types of eccDNAs amplified 1 gene, 103 types of eccDNAs amplified 2 genes, 15 types of eccDNAs amplified 3 genes, 6 types of eccDNAs amplified 4 genes, 10 types of eccDNAs amplified 5 genes, 2 types of eccDNAs amplified 6 genes, 3 types of eccDNAs amplified 7 genes, and 3 types of eccDNAs amplified 8 genes (Fig. 1G).

We further confirmed that the size of eccDNAs among FaDu/DDP cell lines was distributed from 0.01 kb to 1000 kb with two distinctive peaks at 0.1 kb and 0.7 kb (Fig. 1H), and the size of eccDNAs among FaDu cell lines showed a similar pattern (Fig. 1I).

### Identification of differentially expressed eccDNAs and enrichment analysis of the eccDNAs encoding amplified genes

**Fig. 2:**
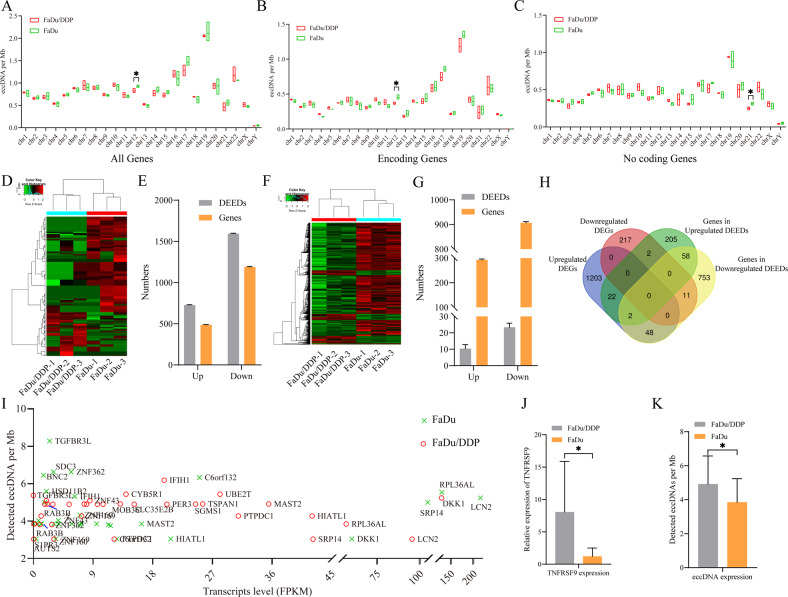
Differentially expressed eccDNAs and genes. **A** Whole genome-wide eccDNA expression is shown by chromosome grouping, as inferred from FaDu/DDP cells compared to FaDu cells. **B** Whole genome-wide eccDNA expression amplifying encoding genes is shown in grouping by chromosome, as inferred from FaDu/DDP cells compared to FaDu cells. **C** Whole genome-wide eccDNA expression amplifying no genes is shown in grouping by chromosome, as inferred from FaDu/DDP cells compared to FaDu cells. **D** Cluster heat map shows the DEEDs between FaDu/DDP cells and FaDu cells. **E** Number of DEED types and amplifying genes in FaDu/DDP cells and FaDu cells. **F** Cluster heat map shows the DEGs between FaDu/DDP cells and FaDu cells. **G** Number of DEED-amplifying complete encoding genes and corresponding encoding genes in FaDu/DDP cells and FaDu cells. **H** Venn diagram shows the significant overlapping genes between the complete encoding genes carried by DEEDs and DEGs. **I** EccDNA counts per gene relative to the average transcription level of FaDu/DDP cells and FaDu cells. **J** Relative mRNA expression (*z* scores) of *TNFRSF9* between FaDu/DDP cells and FaDu cells. **K** Relative eccDNA expression (counts) of [*TNFRSF9*^*circle exon 1-9*^] between FaDu/DDP cells and FaDu cells; *p < 0.05.

Chromosomal 3p arm loss is a frequent genetic event observed in HNSCC [15]. Therefore, we further analyzed the genomic distribution of eccDNA on different chromosomes. Interestingly, we found that gene-rich chromosome 19 contributed to a >2.5-fold higher average frequency of eccDNAs per Mb than other chromosomes, and gene-poor chromosome Y contributed to a more than 10-fold lower average frequency of eccDNAs per Mb than the other chromosomes (Supplementary Table 3). Furthermore, there was no significant difference in the distribution of these eccDNAs between FaDu and FaDu/DDP cells across most of the chromosomes, except for chromosome 12 (Fig. 2A). Then, we further studied the distribution of these eccDNAs on the long arm and the short arm of chromosomes. The results showed that there was no eccDNA located in 13q, 14q, 15q, 22q or Yq. There was no significant difference in the distribution of these eccDNAs between FaDu and FaDu/DDP cells in the long arm and/or short arm of the chromosomes, except for chromosome 1p (Supplementary Fig. 1A–B). Then, we further divided the eccDNAs into two groups: eccDNAs amplified encoding genes, and eccDNAs did not amplify any genes. We found that the distribution of eccDNAs showed a significant difference on chromosomes 11, 12, 14, 16, 20, 21 and 22 in FaDu/DDP cells (Supplementary Fig. 1C). The distribution of eccDNAs showed a significant difference on chromosomes 4, 5, 6, 9, 11, 17, 18, 19 and Y in FaDu cells (Supplementary Fig. 1D). Furthermore, we compared the distribution of these eccDNAs amplifying encoding genes between FaDu/DDP and FaDu cells. We found that there was no significant difference in the distribution of these eccDNAs with encoding genes on different chromosomes, except for chromosome 12 (Fig. 2B). There was no significant difference in the distribution of these eccDNAs, which did not amplify any genes on different chromosomes, except for chromosome 21 (Fig. 2C).

In addition, there were no eccDNAs encoding genes located in 13q, 14q, 15q, 21q, 22q, Yq and Yp (Supplementary Fig. 1E–F). There was no significant difference in the distribution of these eccDNAs, which did not amplify any genes in the long arms and/or short arms of chromosomes, except for chromosomes 11q, 12q, 17q and 17p (Supplementary Fig. 1E–F). Furthermore, there were no eccDNAs that did not amplify any genes located in 13q, 14q, 15q, 22q or Yq (Supplementary Fig. 1G–H). There was no significant difference in the distribution of these eccDNAs, which did not amplify any genes in the long arms and/or short arms of different chromosomes, except for chromosome 7q (Supplementary Fig. 1G–H). These results suggested that the types and distribution of eccDNAs did not differ significantly between FaDu/DDP cells and FaDu cells. Therefore, we wanted to further study whether there was a change in the content of eccDNA between FaDu/DDP and FaDu cells.

To further study the differential expression of eccDNAs between FaDu/DDP and FaDu cells, edgeR (v0.6.9) software was used to perform normalization and differentially expressed eccDNAs (DEEDs) filtering by *p* value and fold change. Eventually, a total of 2307 DEEDs were preliminarily screened with 1163 eccDNA amplified genes with a threshold of |log2FC | >1 and adjusted *p* < 0.05. Compared with the FaDu cells, 720 DEEDs together with 480 genes and 1587 DEEDs together with 1183 genes were upregulated and downregulated in the FaDu/DDP cells, respectively (Fig. 2D, E, Supplementary Table 4).

Furthermore, the GO and pathway enrichment analyses were performed based on the differentially expressed eccDNA-associated encoding genes. The results of the GO analysis revealed that the upregulated DEEDs amplified genes were enriched in CC terms (voltage-gated sodium channel complex, and apical plasma membrane), MF terms (voltage-gated sodium channel activity, and retinoic acid binding) and BP terms (negative regulation of glucuronosyltransferase activity, and negative regulation of cellular glucuronidation). The downregulated DEEDs amplified genes were enriched in CC terms (intracellular, endoplasmic reticulum lumen), MF terms (type I interferon receptor binding, cytokine activity) and BP terms (T cell activation involved in immune response, natural killer cell activation involved in immune response) (Supplementary Fig. 2A–B). The KEGG results showed that both upregulated and downregulated genes played important regulatory roles in the drug resistance-related pathways, such as the insulin signaling pathway, drug metabolism-other enzymes (Supplementary Fig. 2C) and regulation of autophagy (Supplementary Fig. 2D).

### Relationship and combined analysis between the expression profiles of eccDNAs and the expression profiles of encoding gene transcripts amplified on eccDNA

To investigate whether the expression of DEEDs is positively related to the transcript expression level of encoding genes from DEEDs, we first performed whole transcriptome sequencing in FaDu/DDP and FaDu cells. A total of 1509 differentially expressed encoding genes (DEGs) were preliminarily screened by the threshold of |log2FC | >2 and adjusted *p* < 0.05. Compared with the FaDu cells, 1275 and 230 DEGs were upregulated and downregulated in the FaDu/DDP cells, respectively (Fig. 2F, Supplementary Table 5). In addition, 206 and 35 differentially expressed long noncoding RNAs (lncRNAs) were upregulated and downregulated in the FaDu/DDP cells, respectively (Supplementary Fig. 2E, Supplementary Table 6), and 96 and 121 differentially expressed circular RNAs (circRNAs) were upregulated and downregulated in the FaDu/DDP cells, respectively (Supplementary Fig. 2F, Supplementary Table 7).

Then, we further analyzed the relationship between the transcript expression level of encoding genes from the DEEDs and eccDNA frequencies. Considering that the sequences of some encoding genes amplified on DEEDs are not complete while the transcripts obtained by our sequencing are targeted on the full-length transcripts by default, we first screened out the DEEDs that amplified the full-length sequences of encoding genes. As the results showed, 8 upregulated DEEDs amplified 289 full-length encoding genes and 23 downregulated DEEDs amplified 872 full-length encoding genes (Fig. 2G, Supplementary Table 8). We overlapped the full-length encoding genes amplified on DEEDs with DEGs between FaDu/DDP and FaDu cells, and the results showed that 24 upregulated DEGs were amplified on 7 upregulated DEEDs and 11 downregulated DEGs were amplified on 10 downregulated DEEDs (Fig. 2H, Supplementary Table 9). We found a positive correlation between the ratios of eccDNA/MB and encoding genes/MB, which indicated that there were some highly expressed genes totally or partly transcribed from related sequences of eccDNAs, except for genome linear DNA (Fig. 2I). For example, the expression of *TNFRSF9* was significantly increased in FaDu/DDP (Fig. 2J), and the expression of [*TNFRSF9*^*circle exon 1-9*^] was significantly increased in FaDu/DDP (Fig. 2K). These findings indicate that eccDNA frequencies could explain transcription levels.

### eccDNA verification

**Fig. 3:**
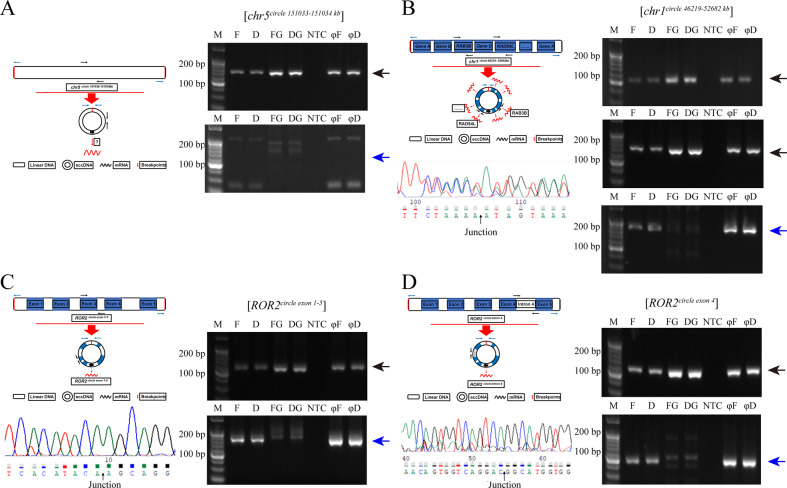
EccDNA validation. **A** Gel images for validated eccDNAs amplified with no gene ([*chr5*^*circle 151033-151034 kb*^]). **B** eccDNA amplified with multiple coding genes ([*chr1*^*circle 46219-52682 kb*^]). **C** eccDNA ([*ROR2*^*circle exon 1-5*^] and (**D**) [*ROR2*^*circle intron 4*^]) by outward PCR (blue arrows), inward PCR (black arrows), gel electrophoresis, schematic diagram and Sanger sequencing. EccDNAs are named according to gene content. blue boxes: exons. M: Marker. F: eccDNA of FaDu cells. D: eccDNA of FaDu/DDP cells. FG: genomic DNA of FaDu cells. DG: genomic DNA of FaDu/DDP cells. φF: phi29 (φ) amplified eccDNA of FaDu cells. φD: phi29 (φ) amplified eccDNA of FaDu/DDP cells. NTC: nontemplate control.

As we previously described, we selected four kinds of eccDNAs for verification: eccDNA amplifying no gene ([*chr5*^*circle 151033-151034 kb*^]), eccDNA amplifying multiple coding genes ([*chr1*^*circle 46219-52682 kb*^]), and one coding gene mapped to two different eccDNAs ([*ROR2*^*circle exon 1-5*^] and [*ROR2*^*circle intron 4*^]). We next verified four different types of eccDNAs via outward PCR, inward PCR and Sanger sequencing. We found that [*chr5*^*circle 151033-151034 kb*^] may not be eccDNA (Fig. 3A, Supplementary Fig. 3A). We confirmed that [*chr1*^*circle 46219-52682 kb*^] is an eccDNA that amplified multiple coding genes, such as RAB3B and RAD54L (Fig. 3B). In addition, we also confirmed that [*ROR2*^*circle exon 1-5*^] may be an eccDNA that amplifies the full-length sequences of ROR2 (Fig. 3C) while [*ROR2*^*circle intron 4*^] may be an eccDNA that amplifies intron 4 of ROR2 (Fig. 3D).

### RAB3B is upregulated in HNSCC and correlates with the prognosis of HNSCC patients

**Fig. 4:**
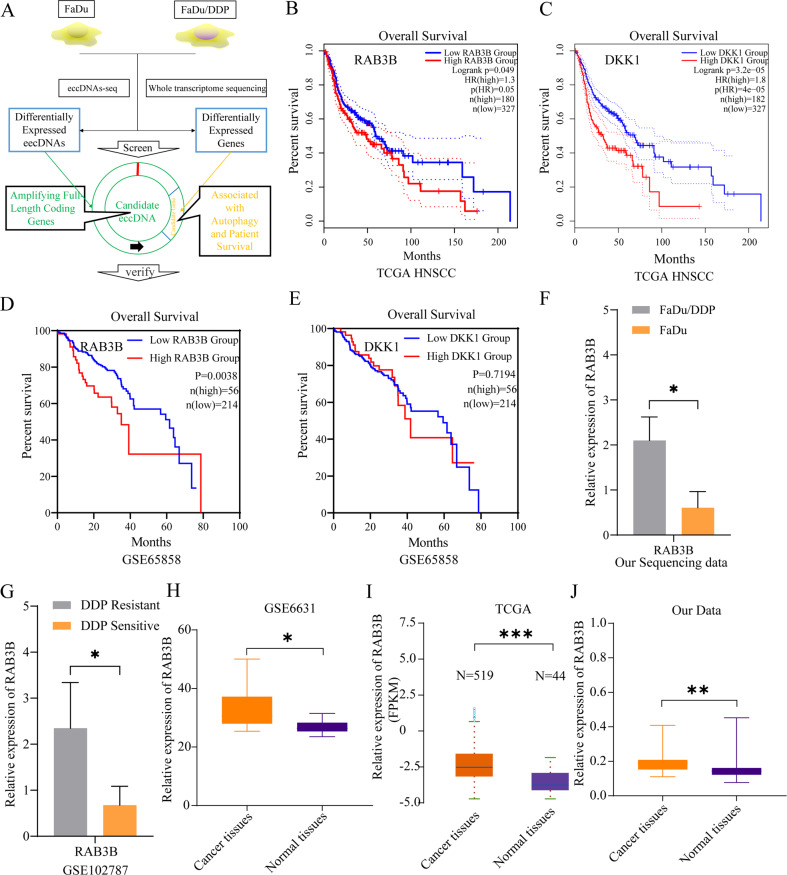
Flowchart of screening out the candidate gene RAB3B followed by further validation based on TCGA and GEO databases. **A** Flowchart of candidate gene for further study. Kaplan–Meier analysis of the OS rate in HNSCC patients in the TCGA database with high or low expression of RAB3B (**B**) and DKK1 (**C**). Kaplan–Meier analysis of the OS rate in HNSCC patients in the GSE658658 database with high or low expression of RAB3B (**D**) and DKK1 (**E**). **F** Relative mRNA expression (*z* scores) of RAB3B between FaDu/DDP cells and FaDu cells in our sequencing data. **G** Relative mRNA expression (*z* scores) of RAB3B between DDP-resistant HNSCC cells and DDP-sensitive HNSCC cells in the GSE102787 database. **H** Relative mRNA expression (*z* scores) of RAB3B between DDP-resistant HNSCC cells and DDP-sensitive HNSCC cells in the GSE102787 database. Flowchart of candidate gene for further study. **I** Relative mRNA expression (*z* scores) of RAB3B between cancer tissues and normal tissues of HNSCC patients in TCGA database. **J** Relative mRNA expression of RAB3B between cancer tissues and normal tissues of HNSCC patients in our database. Data are the means ± SD (*n* = 3 independent experiments), *p < 0.05, **p < 0.01, ***p < 0.001.

To further study the mechanism of eccDNA in regulating DDP resistance, we developed a process for screening drug resistance-related genes and eccDNAs (Fig. 4A). According to this process, we first selected the genes with high expression in Supplementary Table 10 and further analyzed the correlation between these genes and the overall survival of HNSCC patients on GEPIA (http://gepia2.cancer-pku.cn/#survival). We found that only two candidate genes were negatively related to the overall survival (OS) of HNSCC patients, namely, RAB3B and DKK1 (Fig. 4B, C, Supplementary Fig. 4A–U). Similar to the results of TCGA data, we further confirmed that high expression of RAB3B showed poor overall survival in GSE65858 but DKK1 did not share a similar trend (Fig. 4D, E). Compared with DDP-sensitive cells, RAB3B was significantly upregulated in DDP-resistant cells not only in our sequencing data but also in GSE102787 (Fig. 4F, G). DKK1 was not significantly upregulated in DDP-resistant GSE102787 cells (Supplementary Fig. 4V). In addition, we also found that the expression of RAB3B was upregulated in HNSCC tissues compared with related normal tissues (Fig. 4H–J). Therefore, we chose RAB3B and [*chr1*^*circle 46219-52682 kb*^] for further study.

### RAB3B induces autophagy-mediated chemoresistance of FaDu cells in vitro

**Fig. 5:**
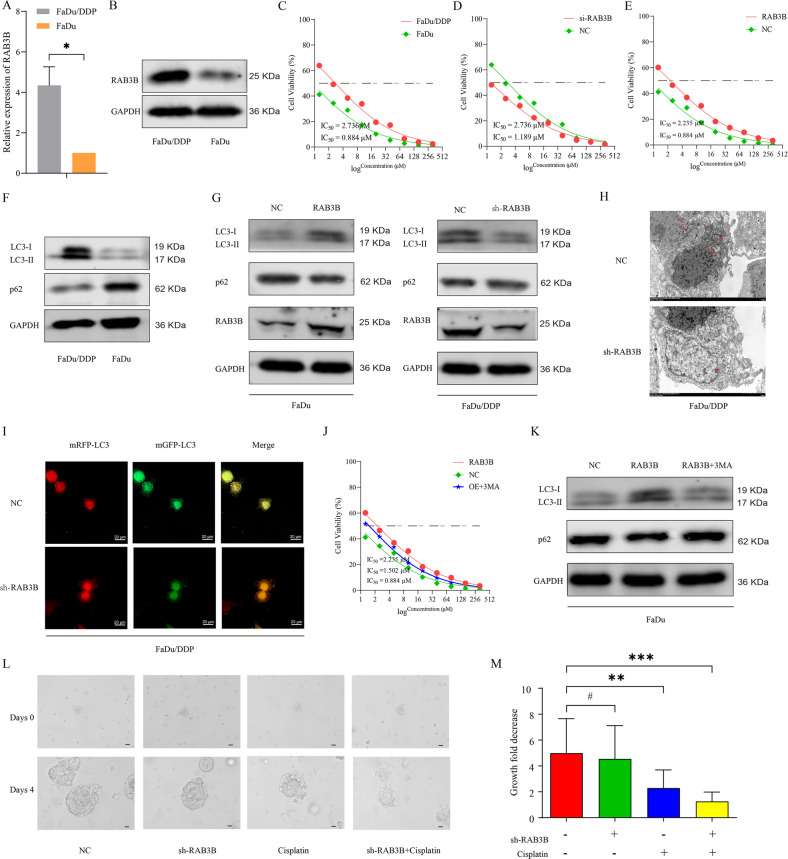
RAB3B could promote the DDP resistance of HSCC by inducing autophagy. **A** Relative mRNA expression of RAB3B in FaDu/DDP cells and FaDu cells was determined by qRT-PCR. **B** Expression of RAB3B in FaDu/DDP cells and FaDu cells was determined by Western blot. **C** Sensitivities of FaDu/DDP cells and FaDu cells under different concentrations of DDP were determined by CCK-8 assay. **D** Sensitivities of FaDu/DDP cells infected with RAB3B shRNA or not under different concentrations of DDP were determined by CCK-8 assay. **E** Sensitivities of FaDu cells transfected with RAB3B overexpression plasmid or not under different concentrations of DDP were determined by CCK-8 assay. **F** Protein levels of LC3B and p62 in FaDu/DDP cells and FaDu cells were determined by Western blot. **G** Protein levels of LC3B and p62 in FaDu/DDP cells infected with RAB3B shRNA or not were determined by Western blot, and the protein levels of LC3B and p62 in FaDu cells transfected with RAB3B overexpression plasmid or not were determined by Western blot. **H** Autophagy was evaluated in FaDu/DDP cells infected with RAB3B shRNA or not using TEM. **I** FaDu/DDP cells stably expressing stubRFP-sensGFP-LC3 under shRNA transfection were observed by fluorescence microscopy. **J** Sensitivities of FaDu cells transfected with RAB3B overexpression plasmid, cotransfected with RAB3B overexpression plasmid and 3MA or not under different concentrations of DDP were determined by CCK-8 assay. **K** Protein levels of LC3B and p62 in FaDu cells transfected with RAB3B overexpression plasmid, cotransfected with RAB3B overexpression plasmid and 3-MA or not were determined by Western blot. **L** Effects of sh-RAB3B and/or DDP on the viability of PDO (scale bar, 100 μm). **M** Measurement of the growth of PDO in response to knockdown of RAB3B and DDP. Data are the means ± SD (*n* = 3 independent experiments); *p < 0.05, **p < 0.01, ***p < 0.001, ^#^*p* > 0.05.

RAB3B, a member of the small GTP-binding protein RAB family that is involved in cell autophagy [16, 17] and drug resistance [18], has been reported as an important oncogene in various cancers [19]. However, its roles in autophagy and drug resistance have not been reported in HSCC. Therefore, we first detected the expression levels of RAB3B in FaDu/DDP and FaDu cells. Compared with FaDu cells, RAB3B was significantly upregulated in FaDu/DDP cells (Fig. 5A, B). To further explore the role of RAB3B in FaDu cells, we transfected FaDu/DDP cells with three individual shRNAs against RAB3B. Then, we selected sh-RAB3B-2 for the subsequent experiments due to its higher inhibition efficiency (Supplementary Fig. 5A–B). Plasmids containing pcDNA-RAB3B were transfected into FaDu cells to upregulate the expression of RAB3B, and the overexpression efficiency was verified (Supplementary Fig. 5C–D). We first confirmed that FaDu/DDP cells were resistant to DDP (Fig. 5C). Then, we found that RAB3B knockdown significantly enhanced the sensitivity of FaDu/DDP cells to DDP (Fig. 5D). RAB3B overexpression heightened the IC_50_ values of FaDu cells to DDP (Fig. 5E). However, we found that RAB3B knockdown or overexpression did not change the proliferation of FaDu/DDP cells (Supplementary Fig. 4E).

Then, we explored whether RAB3B regulates the chemosensitivity of FaDu/DDP cells by regulating autophagy. Compared with FaDu cells, we found that the conversion from LC3-I to LC3-II was promoted while the expression of p62 was downregulated in FaDu/DDP cells (Fig. 5F). The WB results showed that the conversion from LC3-I to LC3-II was inhibited while the expression of p62 was elevated in sh-RAB3B-transfected FaDu/DDP cells, and the opposite phenomenon was observed in RAB3B-overexpressing FaDu cells (Fig. 5G). In addition, we also used TEM to evaluate autophagosomes and detected a substantial decrease in the accumulation of autophagic vesicles in sh-RAB3B-transfected FaDu/DDP cells (Fig. 5H). In addition, we observed that both the numbers of mRFP-LC3 puncta and mGFP-LC3 puncta paralleled the expression levels of RAB3B (Fig. 5I). Furthermore, we found that the treatment of pcDNA-RAB3B-transfected FaDu cells with 3-MA restored chemosensitivity (Fig. 5J). Treatment with 3-MA in pcDNA-RAB3B-transfected FaDu cells reversed the increased expression levels of LC3-II and the decreased expression levels of p62 induced by pcDNA-RAB3B (Fig. 5K). Then, we built PDOs from human tissue of hypopharyngeal cancer patients, and the expression level of RAB3B was higher in cancer tissues than in adjacent normal tissues (Supplementary Fig. 5F). We found that DDP treatment (0.8 μM) caused a substantial reduction in PDO growth (Fig. 5L, M). The combination of RAB3B knockdown and DDP treatment (0.8 μM) caused a more significant reduction in PDO growth than any individual group (Fig. 5L, M). These results suggested that RAB3B can induce autophagy and promote the chemoresistance of FaDu/DDP cells in vitro.

## Discussion

EccDNAs have long been known to exist in normal tissues and cancer tissues [20]. However, the expression and characteristics of eccDNAs in drug-resistant cells have not yet been reported. Here, we thoroughly analyzed the differences in eccDNA expression profiles between FaDu/DDP drug-resistant cells and FaDu parental cells. Although the size and type of eccDNAs did not significantly differ between FaDu/DDP cells and FaDu cells, the number of eccDNAs did significantly differ between these cell types. Then, we selected several representative eccDNAs for verification based on whether the encoding genes were amplified from the eccDNA and the number of encoding genes in the eccDNA. On this basis, the research group combined bioinformatics analysis methods to select and verify the potential mechanism of RAB3B derived from eccDNA in the drug resistance of HSCC. Our study provides a new direction for clinical research on the drug resistance of HSCC.

DNA is packed in the form of linear chromosomes and located in the cell nucleus. Interestingly, an increasing number of researchers have observed smaller lengths of DNA that are from chromosomes that are organized in circular forms. eccDNAs have been referred to as extrachromosomal circular DNA typically smaller than 1 kb [21]. Consistent with previous studies [20, 22], we detected abundant eccDNAs arranged from 0.01 to 1000 kb, with most from 0.1 to 1 kb. To further analyze whether the shedding location of eccDNA is specific in FaDu cells, we classified the shedding location of eccDNAs according to the chromosome. We found that the most common site of eccDNA formation in FaDu cells was chromosome 19, and the least common site was the sex chromosome. However, the chromosome distribution of eccDNAs in FaDu/DDP cells and FaDu cells was only different on chromosome 12. These results indicated that there was no significant difference in the location of eccDNA in FaDu cells during the process of drug resistance. Therefore, we aimed to further study the role and mechanism of the differentially expressed eccDNAs in the DDP resistance of FaDu cells.

By analyzing the differences in gene expression and eccDNA expression between FaDu/DDP cells and FaDu cells, we found that the expression levels of 2307 eccDNAs and 1509 genes were significantly different between FaDu/DDP cells and FaDu cells. Koche RP et al. detected the presence of eccDNAs in neuroblastomas and found 5673 eccDNAs per neuroblastoma; however, there were no significant differences in gene expression derived from eccDNAs, suggesting that eccDNAs may be required but insufficient for oncogene amplification and transcription [23]. Therefore, we focused greater attention on the changes in the expression level and potential molecular mechanisms of eccDNAs amplifying full-length encoding genes during the process of DDP resistance. For example, RAB3B, a gene encoding a small GTP-binding protein that was reported to play a significant role in a variety of cancers, was strongly expressed from amplified eccDNA [24–26]. However, the expression and function of RAB3B in the drug resistance of HNSCC are currently unknown. In our study, we hypothesized and found that RAB3B, which is transcribed from eccDNA with full-length sequences, was an important autophagy regulatory protein and played important roles in the DDP resistance of FaDu cells.

There were several limitations in our study. First, screening and verification of the candidate eccDNA-amplified genes only included the encoding genes and excluded noncoding genes, such as circRNAs, lncRNAs and microRNAs. Second, our results indicated that there were some highly expressed encoding genes with full-length sequences that were totally or partly transcribed from related sequences of eccDNAs, except for genome linear DNA, but we did not prove that the full-length sequences of the encoding genes were directly transcribed from the eccDNAs. Third, the detailed formation mechanisms of eccDNA are still unclear, and further research on the formation mechanism is urgently needed.

## Conclusions

**Fig. 6:**
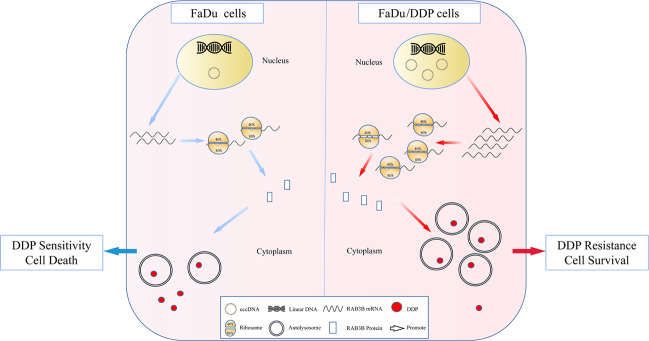
Schematic representation for the role and mechanism of eccDNA in the DDP resistance of FaDu cells. eccDNA combined with linear DNA results in enhancement of DNA amplification, followed higher RNA transcription level, and then more protein translated. RAB3B, which totally or partly transcripted from eccDNA, promoted the DDP resistance of FaDu cells by inducing autophagy.

To the best of our knowledge, this study was first on eccDNA sequencing that focused on the size distribution, chromosome location and expression level of eccDNAs in HSCC. Based on the combined analysis of eccDNA sequencing and encoding gene transcript sequencing, we screened out the encoding gene RAB3B using a full-length sequence and found that it might be transcribed from eccDNA and could play a significant role in DDP resistance by inducing autophagy (Fig. 6).

## Supplementary information

Supplementary materials and methods

Reproducibility Checklist

Supplementary Figure 1

Supplementary Figure 2

Supplementary Figure 3

Supplementary Figure 4

Supplementary Figure 5

Supplementary Table 1-11

## Data Availability

All data are available in the main text or the Supplementary Materials.
