# Supplementary material for: Encoding gene RAB3B exists in linear chromosomal and circular extrachromosomal DNA and contributes to cisplatin resistance of hypopharyngeal squamous cell carcinoma via inducing autophagy
Source: Cell Death Dis. 2022 Feb 22;13(2):171. doi: 10.1038/s41419-022-04627-w (PMC8863882; doi:10.1038/s41419-022-04627-w)
Supplement: Supplementary file 1 — Supplementary materials and methods [file 41419_2022_4627_MOESM1_ESM.docx]

**Supplemental Materials and Methods**

**Patient and tissue specimens**

The study was approved by the ethics committee of the Third Xiangya Hospital of Central South University. Written informed consent was obtained from all patients. The study included 26 patients who was first diagnosed with hypopharyngeal carcinoma and underwent surgery at the Third Xiangya Hospital of Central South University.

**Cell lines and cell culture**

The human HSCC cell line FaDu was purchased from the Chinese Academy of Science (Shanghai, China). FaDu cells were cultured in McCoy’s 5A medium (HyClone, USA) containing 10% fetal bovine serum (Gibco, USA), 100 U/ml penicillin and 100 μg/ml streptomycin (Vicmed, China). FaDu/DDP cells were established and verified by our laboratory. The detailed methods for inducing drug resistance were introduced in our previous papers(1, 2). All cells were cultured at 37 °C in a humidified incubator with 5% CO_2_. 3-Methyladenine (3-MA, MCE, USA) at 10 mM was used to treat the cells.

**Whole transcriptome sequencing**

For the gene expression analyses, total RNA from the FaDu and FaDu/DDP cells was extracted, and RNA libraries were constructed using the TruSeq Stranded total RNA LT Sample Prep Kit (Illumina) with 500 ng total RNA according to the manufacturer’s instructions. The whole transcriptome sequencing service was provided by CloudSeq Biotech Inc. (Shanghai, China).

**Short hairpin RNA (shRNA) and plasmid construction, plasmid transfection and RNA interference**

Full-length RAB3B was synthesized by GENEWIZ and cloned into the pcDNA3.1 vector (Invitrogen). The shRNAs for RAB3B were obtained from RiboBio Co., Ltd. (Guangzhou, China). FaDu cells were infected with shRNA using siLentFect lipid reagent (Bio-Rad, USA) and transfected with plasmid (GeneChem, China) using Lipofectamine 3000 transfection reagent (Thermo Fisher, USA) according to the manufacturer’s protocol. The transfection effects were verified by quantitative real-time PCR assays and Western blotting.

**Quantitative real-time PCR assays**

Quantitative real-time PCR assays (qRT-PCR) were performed as described in our previous study(3). In brief, total RNA from cells and tissues was extracted using TRIzol Reagent (Invitrogen, Carlsbad, CA, USA), and cDNA was synthesized using ReverTra Ace qPCR RT Master Mix (TOYOBO, Osaka, Japan) according to the manufacturer’s instructions. Quantitative real-time PCR (qRT-PCR) was performed using SYBR Green Real-time PCR Master Mix (TOYOBO). Each sample was quantified in triplicate, and the experiment was repeated three times. An analysis was performed using the 2^-∆∆Ct^ method, with GAPDH as the endogenous control. All primer pairs were purchased from Sangon Biotech (Shanghai, China), and all primer sequences are provided in Supplementary Table 11.

**Western blot assays**

Western blot (WB) assays were performed as described in our previous study (3). In brief, whole-cell lysates were prepared in 1× RIPA buffer (KeyGEN) containing 1% PMSF (KeyGEN). The proteins were separated by sodium dodecyl sulfate-polyacrylamide gel electrophoresis (SDS-PAGE), transferred to polyvinylidene fluoride membranes (Millipore), blocked with 5% skim milk for 1 h at room temperature, immunoblotted with primary antibodies overnight and secondary antibodies for 1 h, and visualized on an Odyssey CLx Infrared Imaging System (LI-COR Biosciences, NE, USA). Antibody against RAB3B was purchased from Abcam, antibody against LC3B was purchased from Cell Signaling Technology, and antibodies against p62 and GAPDH were purchased from Proteintech.

**Chemosensitivity assays in vitro**

Chemosensitivity assays were performed as described in our previous study(4). In brief, FaDu or FaDu/DDP cells with or without transfection were seeded into 6-well plates, and medium containing different concentrations of DDP with or without the autophagy inhibitor 3-MA was added. After incubation for 48 h, the absorbance (450 nm) was assessed by water-soluble tetrazolium salt assay using the Cell Counting Kit-8 according to the manufacturer’s protocol. Then, we drew a cell growth curve and calculated the 50% inhibition of growth (IC_50_) value of each cell line for each drug.

**Live-cell imaging for autophagic flux**

Live-cell imaging for autophagic flux was performed as described in a previous study(5). Briefly, mRFP-GFP-LC3 adenoviral particles were purchased from HanBio (Shanghai, China). Cells were infected with adenoviral particles for 24 h. Imaging was performed using an UltraVIEW VoX 3D Live Cell Imaging System and was analyzed by Volocity Demo software (v5.4). All image acquisition settings were maintained in the same state during the image collection.

**Transmission electron microscopy (TEM)**

TEM was performed as described in a previous study(6). Briefly, after transfection with sh-RAB3B, the cells were immediately fixed with 2.0% glutaraldehyde in 0.1 M sodium cacodylate buffer, pH 7.4, and then postfixed in 1% osmium tetroxide, dehydrated in ethanol and embedded in Epon. Then, the samples were examined with a HITACHI H600 transmission electron microscope operated at 80 KV.

**Patient-derived organoid**

The generation of patient-derived organoids (PDOs) was performed as described in a previous study(7). Briefly, tumor tissue was obtained from surgical resections of patients with hypopharyngeal carcinoma, cut into 1-3 mm^3^ pieces and digested with 200 U/ml collagenase (Sigma) and 100 U/ml hyaluronidase (Sigma). The PDOs were mixed with growth factor-reduced Matrigel and cultured in 24-well plates.

PDOs were transiently transfected with a lentivirus expressing sh-RAB3B (GenePharma, China) or incubated with DDP (0.8 μM) for 4 days (8). Paired DIC images for each PDO were acquired on day 1 and day 4 after treatment(9) and used to analyze the growth of organoids. The evaluation of PDO growth was performed following a previously described method(10).

**Bioinformatics analysis**

A gene expression analysis was carried out using the starBase database v3.0 project (<http://starbase.sysu.edu.cn)>(11). The survival analysis was carried out using the Gene Expression Profiling Interaction Analysis database (<http://gepia.cancer-pku.cn)>(12).

**Supplementary Figure Legends**

**Supplementary Figure 1.**

The expression of eccDNAs located on the long arm of the chromosome **(A)** and short arm of the chromosome **(B)** are shown in grouping by chromosome, as inferred from FaDu/DDP cells compared to FaDu cells. The expression of eccDNAs between eccDNAs amplifying coding genes and eccDNAs amplifying no genes in grouping by chromosome was compared, as inferred from FaDu/DDP cells **(C)** and FaDu cells **(D)**. The expression of eccDNAs amplifying encoding genes located on the long arm of chromosome **(E)** and short arm of chromosome **(F)** are shown in groupings by chromosome, as inferred from FaDu/DDP cells compared to FaDu cells. The expression of eccDNAs amplifying no genes located on the long arm of chromosome **(G)** and short arm of chromosome **(H)** are shown in groupings by chromosome, as inferred from FaDu/DDP cells compared to FaDu cells. **p*<0.05, ^#^*p*>0.05.

**Supplementary Figure 2.**

GO enrichment analysis of upregulated DEED-associated genes **(A)** and downregulated DEED-associated genes **(B)**. KEGG enrichment analysis of upregulated DEED-associated genes **(C)** and downregulated DEED-associated genes **(D)**. The cluster heat map shows the differential expression of lncRNAs **(E)** and circRNAs **(F)** between FaDu/DDP cells and FaDu cells.

**Supplementary Figure 3**

Sanger sequencing of integration breakpoints for eccDNAs amplified no ([*chr5^circle 151033-151034 kb^*]). The red highlighted sequence represents the breakpoints of eccDNAs, and the underlined sequence represents the overlapping sequence between the predicted sequence and sequencing sequence.

**Supplementary Figure 4**

**(A-U)** Kaplan-Meier analysis of the OS rate in HNSCC patients in the TCGA database with high or low expression of candidate genes. **(V)** Relative mRNA expression (z-scores) of DKK1 between DDP-resistant HNSCC cells and DDP-sensitive HNSCC cells in the GSE102787 database; ^#^*p*>0.05.

**Supplementary Figure 5**

**(A)** qRT-PCR was used to examine the expression of RAB3B after overexpression in FaDu/DDP cells. **(B)** Western blot analysis of the expression of RAB3B after overexpression in FaDu cells. **(C)** qRT-PCR was used to examine the expression of RAB3B after knockdown in FaDu/DDP cells. **(D)** Western blot analysis of the expression of RAB3B after knockdown in FaDu/DDP cells. **(E)** CCK-8 assay showed the effects of RAB3B knockdown and RAB3B overexpression on proliferation in FaDu/DDP cells. **(F)** qRT-PCR was used to detect the expression of RAB3B in donor patient tumor tissue. Data are the means ± SD (n = 3 independent experiments); * *p*<0.05, ** *p*<0.01, *** *p*<0.001, ^#^ *p*>0.05.

**Reference**

1. Tan PY, Chang CW, Chng KR, Wansa KD, Sung WK, Cheung E. Integration of regulatory networks by NKX3-1 promotes androgen-dependent prostate cancer survival. Mol Cell Biol. 2012;32(2):399-414.

2. Peng X, Li W, Tan G. Reversal of taxol resistance by cisplatin in nasopharyngeal carcinoma by upregulating thromspondin-1 expression. Anticancer Drugs. 2010;21(4):381-8.

3. Li D, Lin C, Li N, Du Y, Yang C, Bai Y, et al. PLAGL2 and POFUT1 are regulated by an evolutionarily conserved bidirectional promoter and are collaboratively involved in colorectal cancer by maintaining stemness. EBioMedicine. 2019;45:124-38.

4. Wang X, Li H, Li W, Xie J, Wang F, Peng X, et al. The role of Caspase-1/GSDMD-mediated pyroptosis in Taxol-induced cell death and a Taxol-resistant phenotype in nasopharyngeal carcinoma regulated by autophagy. Cell Biol Toxicol. 2020;36(5):437-57.

5. Hua F, Li K, Yu JJ, Lv XX, Yan J, Zhang XW, et al. TRB3 links insulin/IGF to tumour promotion by interacting with p62 and impeding autophagic/proteasomal degradations. Nat Commun. 2015;6:7951.

6. Cai Q, Wang S, Jin L, Weng M, Zhou D, Wang J, et al. Long non-coding RNA GBCDRlnc1 induces chemoresistance of gallbladder cancer cells by activating autophagy. Mol Cancer. 2019;18(1):82.

7. Driehuis E, Kretzschmar K, Clevers H. Establishment of patient-derived cancer organoids for drug-screening applications. Nat Protoc. 2020;15(10):3380-409.

8. Arena S, Corti G, Durinikova E, Montone M, Reilly NM, Russo M, et al. A Subset of Colorectal Cancers with Cross-Sensitivity to Olaparib and Oxaliplatin. Clin Cancer Res. 2020;26(6):1372-84.

9. Xiao L, Wu J, Wang JY, Chung HK, Kalakonda S, Rao JN, et al. Long Noncoding RNA uc.173 Promotes Renewal of the Intestinal Mucosa by Inducing Degradation of MicroRNA 195. Gastroenterology. 2018;154(3):599-611.

10. Jiao Z, Cai H, Long Y, Sirka OK, Padmanaban V, Ewald AJ, et al. Statin-induced GGPP depletion blocks macropinocytosis and starves cells with oncogenic defects. Proc Natl Acad Sci U S A. 2020;117(8):4158-68.

11. Li JH, Liu S, Zhou H, Qu LH, Yang JH. starBase v2.0: decoding miRNA-ceRNA, miRNA-ncRNA and protein-RNA interaction networks from large-scale CLIP-Seq data. Nucleic Acids Res. 2014;42(Database issue):D92-7.

12. Tang Z, Li C, Kang B, Gao G, Li C, Zhang Z. GEPIA: a web server for cancer and normal gene expression profiling and interactive analyses. Nucleic acids research. 2017;45(W1):W98-W102.
