# Supplementary figures and images for: Encoding gene RAB3B exists in linear chromosomal and circular extrachromosomal DNA and contributes to cisplatin resistance of hypopharyngeal squamous cell carcinoma via inducing autophagy
Source: Cell Death Dis. 2022 Feb 22;13(2):171. doi: 10.1038/s41419-022-04627-w (PMC8863882; doi:10.1038/s41419-022-04627-w)

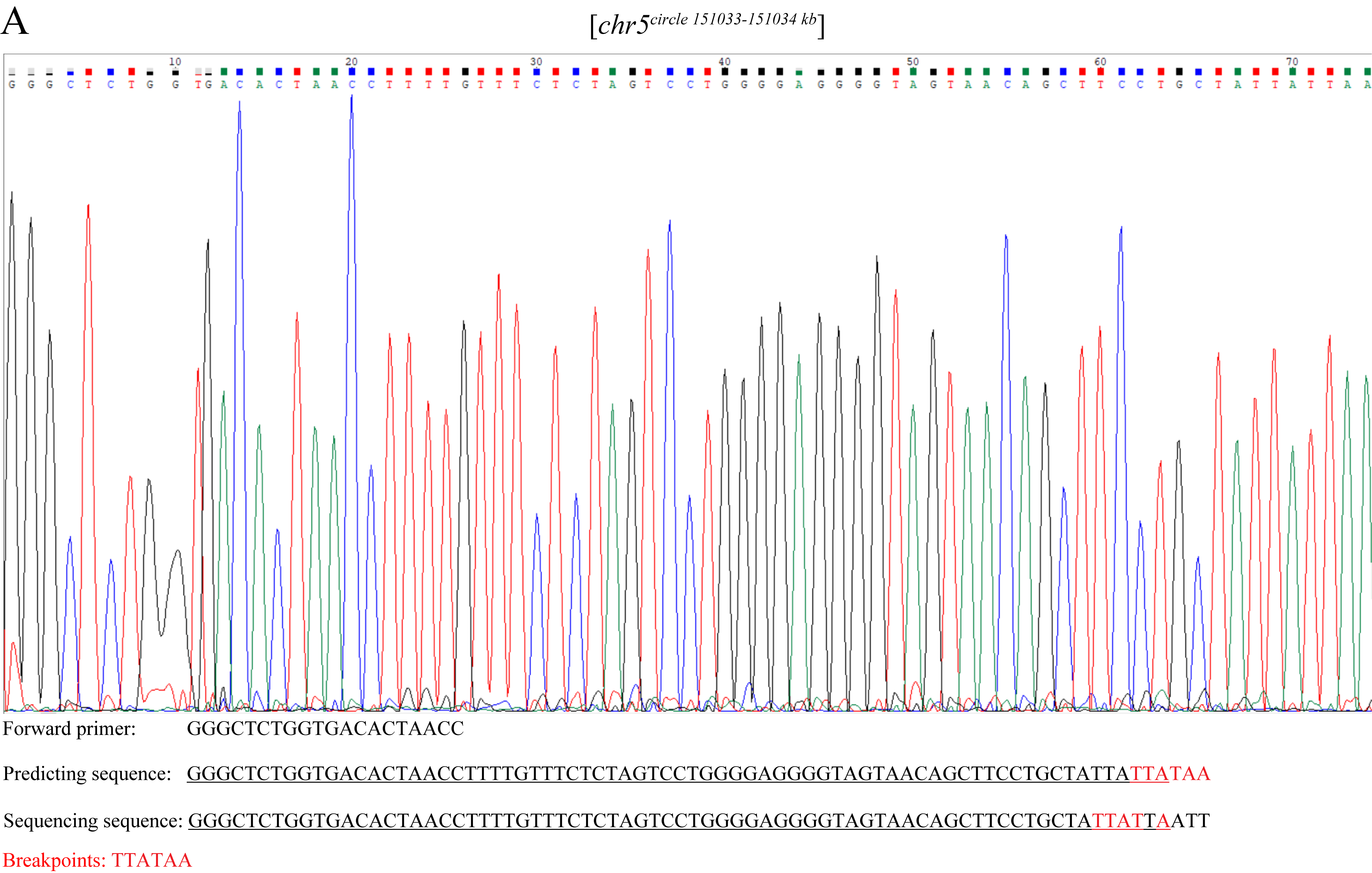

Supplement: Supplementary file 5 — Supplementary Figure 3 [file 41419_2022_4627_MOESM5_ESM.tif]
